# Structure of equations for gravity mass flows with entrainment

**DOI:** 10.1038/s41467-024-48605-6

**Published:** 2024-05-30

**Authors:** Dieter Issler, Peter Gauer, Callum Tregaskis, Hervé Vicari

**Affiliations:** 1 Norwegian Geotechnical Institute, P.O. Box 3930 Ullevål Stadion, 0806 Oslo, Norway; 2 Present Address: WSL Institute for Snow and Avalanche Research SLF, 7260 Davos Dorf, Switzerland

**Keywords:** Natural hazards, Applied mathematics, Geomorphology, Civil engineering

**arising from** S. P. Pudasaini & M. Krautblatter *Nature Communications* 10.1038/s41467-021-26959-5 (2021)

Entrainment of bed material may greatly increase the moving mass of gravity mass flows (GMFs) and thus strongly influence their dynamics, but this process is notoriously difficult to model and often neglected. Pudasaini and Krautblatter^1^ (henceforth denoted by PK) claimed in this journal that all earlier depth-averaged GMF models omit an important inertial effect due to entrainment and derived far-reaching consequences for GMF mobility from a modified equation of motion. We show that the modified equation violates energy conservation and identify an error in its derivation. Defining the system boundaries consistently, one recovers energy conservation and the standard form of the equation of motion in the presence of entrainment or deposition.

## The issue

PK work in the context of 2D depth-averaged two-phase models, but the issue arises also in 1D one-phase models. Denote time by *t*, distance along the flow path by *s*, and, for simplicity, assume the flow and bed density, *ρ*, are uniform and equal. GMFs typically being thin, the bed-normal velocity *w*(*s*, *z*, *t*) is neglected and the longitudinal velocity *u*(*s*, *z*, *t*) is approximated by $\bar{u}(s,\, t)$, its average value across the flow depth *h*(*s*, *t*). If the mass is entrained from the bed at the rate *q*_*e*_(*s*, *t*), the mass balance of a flow slice normal to the bed is expressed as

1 $${\partial }_{t}( \, \rho h)+{\partial }_{s}(\rho h\bar{u})={q}_{e}.$$

After repeated debates, e.g., refs. ^2–5^, a broad consensus has been reached that the momentum balance equation has the general form

2 $${\partial }_{t}( \, \rho h\bar{u})+{\partial }_{s}\left(\rho h{\bar{u}}^{2}-h{\bar{\sigma }}_{ss}\right)=\rho {g}_{s}h-{\tau }_{b}+{q}_{e}{u}_{b}.$$

*g*_*s*_ is the downslope component of gravity, ${\bar{\sigma }}_{ss}$ the depth-averaged longitudinal stress, *τ*_*b*_ the basal shear stress. The term + *q*_*e*_*u*_*b*_ accounts for the momentum influx due to the initial velocity *u*_*b*_ of the eroded material. For 1D single-phase flows, PK’s equations (PK.1) and (PK.2) simplify to Eqs. (1) and (2).

From Eqs. (1) and (2), one finds the equation of motion for the acceleration $\bar{a}$,

3 $$\bar{a}\equiv ({\partial }_{t}+\bar{u}{\partial }_{s})\bar{u}={g}_{s}+\frac{1}{\rho h}\left[{\partial }_{s}(h{\bar{\sigma }}_{ss})-{\tau }_{b}+(k{u}_{b}-\bar{u}){q}_{e}\right],$$

with *k* = 1. PK contest the legitimacy of this substitution operation, posit *k* = 2 in Eq. (PK.11) and derive far-reaching consequences for GMF mobility from this modification if *u*_*b*_ > 0.

## Energy conservation

**Fig. 1:**
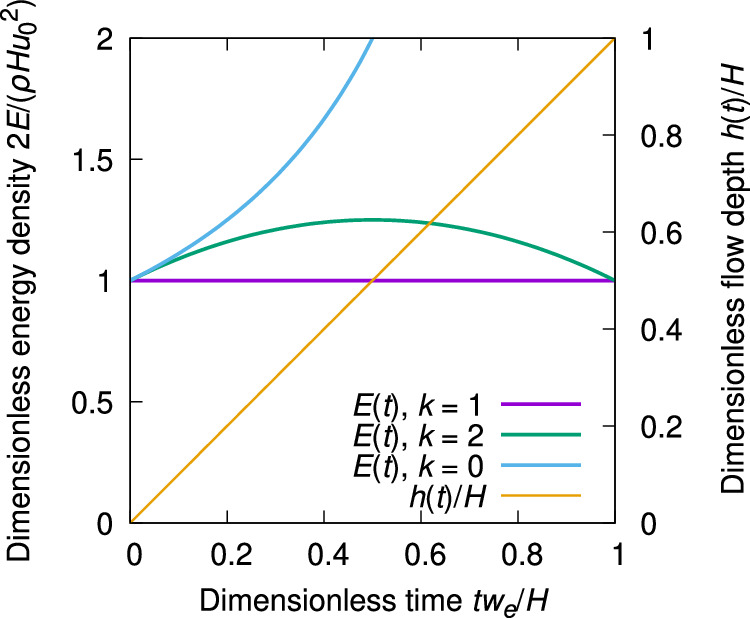
Time evolution of the depth-averaged energy density in the system described by Eq. (4) for different values of *k.* Energy is conserved only for *k* = 1.

We first show that *k* ≠ 1 violates energy conservation even in a purely kinematic setting. Let an inviscid fluid in an infinitely long channel of depth *H* flow at constant uniform speed *u*_0_ under zero gravity and zero stress. Imagine a *virtual* interface moving downward through the channel depth at speed *w*_*e*_ = *q*_*e*_/*ρ*, dividing the fluid into an upper part of depth *h*(*t*) = *w*_*e*_*t* and velocity *u*(*t*), and a lower part of depth *b*(*t*) = *H* − *w*_*e*_*t* and velocity *v*(*t*). This artificial division does not change the flow, thus *u*(*t*) ≡ *v*(*t*) ≡ *u*_0_ = cst. With the initial conditions, Eqs. (1) and (2) handle this problem correctly and naturally. The equation of motion (3) becomes

4 $$\frac{{{{{{{{\rm{d}}}}}}}}u}{{{{{{{{\rm{d}}}}}}}}t}=\frac{(kv-u){w}_{e}}{{w}_{e}t}=\frac{kv-u}{t}\qquad {{{{{{{\rm{and}}}}}}}}\qquad \frac{{{{{{{{\rm{d}}}}}}}}v}{{{{{{{{\rm{d}}}}}}}}t}=\frac{(1-k)v{w}_{e}}{H-{w}_{e}t}$$

for the two layers. One can readily find analytical solutions for any *k* ≠ 0 and calculate the depth-averaged kinetic energy density, *E*, in terms of the dimensionless time $\hat{t}=t{w}_{e}/H$, see Fig. 1:

5 $$u(\hat{t})={u}_{0}\,\frac{1-{(1-\hat{t})}^{k}}{\hat{t}}\qquad {{{{{{{\rm{and}}}}}}}}\qquad v(\hat{t})={u}_{0}{(1-\hat{t})}^{k-1}.$$

Only *k* = 1 gives the correct solution and conserves the kinetic energy, see Fig. 1.

## Origin of the discrepancy

**Fig. 2:**
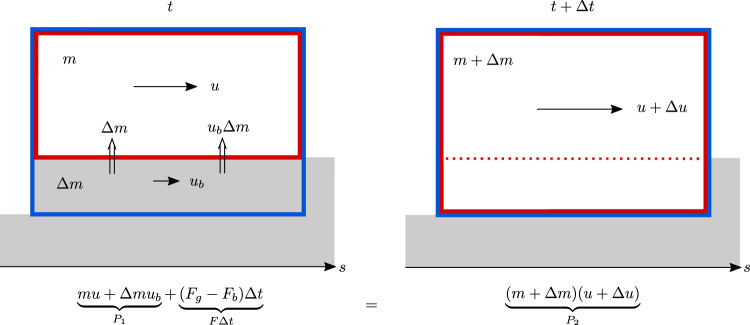
Schematic representation of a control volume. Equations (PK.5) and (PK.6) describe the mechanical system inside the blue rectangle, consisting of the flow and the bed layer that is entrained during the time interval Δ*t*. In contrast, Eqs. (PK.1) and (PK.2) apply only to the flow (red rectangle). The mass and momentum flux from entrainment (double arrows) enters the flow across the bed–flow interface but is internal to the system (bed + flow).

To locate the origin of PK’s factor *k* = 2, consider the momentum balance in a control volume *V*(*t*), whose boundary, ∂*V*(*t*), has a unit outward normal vector $\hat{{{{{{{{\boldsymbol{n}}}}}}}}}$ and moves with velocity ***ω***(*s*, *z*, *t*) [ref. ^6^, ch. 3] (Fig. 2):

6 $$\frac{{{{{{\mathrm{d}}}}}}}{{{{{{\mathrm{d}}}}}} t} \iint_{ V} \rho {{{{{\boldsymbol{u}}}}}}\,{{{{{\mathrm{d}}}}}} V=\underbrace{ \oint_{\partial V} \left[ {{{{{\boldsymbol{\sigma}}}}}} - \rho {{{{{\boldsymbol{u}}}}}} ({{{{{\boldsymbol{u}}}}}} - {{{{{\boldsymbol{\omega}}}}}}) \right] \cdot {\hat{{{{{{\boldsymbol{n}}}}}}}}\,{{{{{\mathrm{d}}}}}} l}_{{I_{{{{{{\mathrm{front}}}}}}}+I_{{{{{{\mathrm{back}}}}}}}+I_{{{{{{\mathrm{top}}}}}}}+I_{{{{{{\mathrm{bed}}}}}}}}} \,+\iint_{V} \rho {{{{{\boldsymbol{g}}}}}}\,{{{{{\mathrm{d}}}}}} V .$$

The critical issue is how to evaluate *I*_bed_, the integral along the lower edge of length Δ*s*. The system described by Eqs. (3) with *k* = 1, (2) and (PK.2) corresponds to the red rectangles in Fig. 2, where the bed–flow interface is the lower boundary and moves at speed *ω*_*z*_ = − *q*_*e*_/*ρ*. This gives a contribution ${I}_{{{{{{{{\rm{red}}}}}}}}}=(-{\tau }_{b}^{{{{{{{{\rm{red}}}}}}}}}+{u}_{b}{\omega }_{z})\Delta s$, the second term quantifying particle-borne momentum influx due to entrainment. In contrast, Eqs. (PK.5)–(PK.10) correctly describe the “blue” system in Fig. 2: the mass entrained during the interval Δ*t* and its momentum *q*_*e*_*u*_*b*_Δ*s*Δ*t* are already contained in the system at time *t*, hence *ω*_*z*_ = 0 and ${I}_{{{{{{{{\rm{blue}}}}}}}}}=-{\tau }_{b}^{{{{{{{{\rm{blue}}}}}}}}}\Delta s$. Note that inertial effects imply ${\tau }_{b}^{{{{{{{{\rm{blue}}}}}}}}} \, < \, {\tau }_{b}^{{{{{{{{\rm{red}}}}}}}}}$ if *u*_*b*_ > 0.

In the last step, PK substitute the r.h.s. of the “red” Eq. (PK.4) for the sum of external and body forces, *F*, in the “blue” Eq. (PK.10) to obtain Eq. (PK.11). While (PK.10) is correct if *ω*_*z*_ = 0, (PK.4) is established for *ω*_*z*_ = − *q*_*e*_/*ρ*. This switch between systems amounts to counting the particle-borne momentum influx due to entrainment twice, hence the erroneous factor *k* = 2. If one consistently uses either the “red” or the “blue” system, the standard form of the equation of motion with *k* = 1 results.

## Enhanced mobility of entraining GMFs

PK’s explanation of the enhanced mobility of entraining GMFs hinges critically on *k* = 2. However, an often overlooked aspect of erosion may facilitate long runout. Cohesive materials are eroded when the shear stress exceeds the peak strength of the bed, *τ*_*c*_. This caps the bed shear stress, ${\tau }_{b}=\min ({\tau }_{f},\, {\tau }_{c})$, however large the shear stress inside the flow, *τ*_*f*_, may be. The difference *τ*_*f*_ − *τ*_*c*_ is available to accelerate the eroded mass from *u*_*b*_ to $\bar{u}$ across an “entrainment layer”, which depth-averaged models contract to an interface with velocity and shear-stress discontinuities. This allows us to estimate^7^

7 $${q}_{e} \sim \frac{\max (0,\, {\tau }_{f}-{\tau }_{c})}{\bar{u}-{u}_{b}}.$$

In summary, we identified an inadvertent switch from one system definition to another as the reason why PK found the initial velocity of entrained mass, *u*_*b*_, to reduce flow inertia at twice the established (and correct) rate. PK’s formulation does not conserve energy and may produce nonphysical results in numerical GMF models. The remaining challenge is to find the functional form of *q*_*e*_ for different types of GMFs.

## References

[1] Pudasaini SP, Krautblatter M (2021). The mechanics of landslide mobility with erosion. Nat. Commun..

[2] Cannon SH, Savage WZ (1988). A mass-change model for the estimation of debris-flow runout. J. Geol..

[3] Hungr O (1990). A mass-change model for the estimation of debris-flow runout: a discussion. J. Geol..

[4] Cannon SH, Savage WZ (1990). A mass-change model for the estimation of debris-flow runout: a reply. J. Geol..

[5] Erlichson H (1991). A mass-change model for the estimation of debris-flow runout, a second discussion: conditions for the application of the rocket equation. J. Geol..

[6] Chadwick, P. *Continuum Mechanics: Concise Theory and Problems*. 2nd edn (Dover Publications, Mineola, N.Y., U.S.A., 1999).

[7] Norem, H. & Schieldrop, B. Stress analyses for numerical modelling of submarine flowslides. NGI Report 522090-10. https://ngi.brage.unit.no/ngi-xmlui/handle/11250/3125578 (Norges Geotekniske Institutt, Oslo, Norway, 1991).

